# ADHD symptoms and diagnosis in adult preterms: systematic review, IPD meta-analysis, and register-linkage study

**DOI:** 10.1038/s41390-021-01929-1

**Published:** 2022-01-07

**Authors:** Rachel Robinson, Polina Girchenko, Anna Pulakka, Kati Heinonen, Anna Lähdepuro, Marius Lahti-Pulkkinen, Petteri Hovi, Marjaana Tikanmäki, Peter Bartmann, Aulikki Lano, Lex W. Doyle, Peter J. Anderson, Jeanie L. Y. Cheong, Brian A. Darlow, Lianne J. Woodward, L. John Horwood, Marit S. Indredavik, Kari Anne I. Evensen, Neil Marlow, Samantha Johnson, Marina Goulart de Mendonca, Eero Kajantie, Dieter Wolke, Katri Räikkönen

**Affiliations:** 1 University of Helsinki, Helsinki, Finland; 2 Finnish Institute for Health and Welfare, Helsinki, Finland; 3 Faculty of Social Sciences, Tampere University, Tampere, Finland; 4 Department of Neonatology, University of Bonn, Bonn, Germany; 5 Children’s Hospital, University of Helsinki and Helsinki University Hospital, Helsinki, Finland; 6 Royal Women’s Hospital, Melbourne, VIC Australia; 7 University of Melbourne, Melbourne, VIC Australia; 8 Murdoch Children’s Research Institute, Melbourne, VIC Australia; 9 Turner Institute for Brain and Mental Health, School of Psychological Sciences, Monash University, Clayton, VIC Australia; 10 Department of Paediatrics, University of Otago, Christchurch, New Zealand; 11 University of Canterbury, Christchurch, New Zealand; 12 Department of Clinical and Molecular Medicine, Norwegian University of Science and Technology, Trondheim, Norway; 13 Department of Physiotherapy, Oslo Metropolitan University, Oslo, Norway; 14 University College London, London, UK; 15 University of Leicester, Leicester, UK; 16 University of Warwick, Coventry, UK; 17 Department of Neuroscience, Psychology and Behaviour, School of Psychology, University of Leicester, Leicester, UK; 18 PEDEGO Research Unit, MRC Oulu, Oulu University Hospital and University of Oulu, Oulu, Finland

## Abstract

**Background:**

This study examined differences in ADHD symptoms and diagnosis between preterm and term-born adults (≥18 years), and tested if ADHD is related to gestational age, birth weight, multiple births, or neonatal complications in preterm borns.

**Methods:**

(1) A systematic review compared ADHD symptom self-reports and diagnosis between preterm and term-born adults published in PubMed, Web of Science, and PROQUEST until April 2021; (2) a one-stage Individual Participant Data(IPD) meta-analysis (*n* = 1385 preterm, *n* = 1633 term; born 1978–1995) examined differences in self-reported ADHD symptoms[age 18–36 years]; and (3) a population-based register-linkage study of all live births in Finland (01/01/1987–31/12/1998; *n* = 37538 preterm, *n* = 691,616 term) examined ADHD diagnosis risk in adulthood (≥18 years) until 31/12/2016.

**Results:**

Systematic review results were conflicting. In the IPD meta-analysis, ADHD symptoms levels were similar across groups (mean *z*-score difference 0.00;95% confidence interval [95% CI] −0.07, 0.07). Whereas in the register-linkage study, adults born preterm had a higher relative risk (RR) for ADHD diagnosis compared to term controls (RR = 1.26, 95% CI 1.12, 1.41, *p* < 0.001). Among preterms, as gestation length (RR = 0.93, 95% CI 0.89, 0.97, *p* < 0.001) and SD birth weight *z*-score (RR = 0.88, 95% CI 0.80, 0.97, *p* < 0.001) increased, ADHD risk decreased.

**Conclusions:**

While preterm adults may not report higher levels of ADHD symptoms, their risk of ADHD diagnosis in adulthood is higher.

**Impact:**

- Preterm-born adults do not self-report higher levels of ADHD symptoms, yet are more likely to receive an ADHD diagnosis in adulthood compared to term-borns.
- Previous evidence has consisted of limited sample sizes of adults and used different methods with inconsistent findings. This study assessed adult self-reported symptoms across 8 harmonized cohorts and contrasted the findings with diagnosed ADHD in a population-based register-linkage study.
- Preterm-born adults may not self-report increased ADHD symptoms. However, they have a higher risk of ADHD diagnosis, warranting preventive strategies and interventions to reduce the presentation of more severe ADHD symptomatology in adulthood.

## Background

Individuals born preterm (<37 + 0 wks + days gestational age) have an increased risk for attention-deficit hyperactivity disorder (ADHD) and elevated symptom levels in childhood.^1^ This increased risk not only characterizes tshose born very preterm(VP; <32 + 0 wks + days) or with very low birth weight (VLBW; <1500 g), but the risk increases linearly with each declining week of gestation.^1^

Although well established in childhood, there is conflicting evidence as to whether the risk for ADHD persists in preterm adults. The conflicting information may reflect the varying quality of evidence, diverse methods, and small sample sizes of the cohort studies with adult follow-ups,^2,3^ whereas the population-based register-linkage studies have examined ADHD diagnosis in samples including both children and adults.^1,4^

Studying ADHD in preterm borns beyond childhood is important as the prevalence rate of ADHD diagnosis in the general population declines with age.^5,6^ Moreover, not all children with ADHD diagnosis in childhood continue to meet the diagnostic criteria in adulthood and the symptom profiles may change with inattention showing the highest and impulsivity-hyperactivity the lowest continuity.^7^ Also, the predominately male presentation of ADHD in childhood may no longer be present in adulthood.^6^

We conducted a systematic review of existing studies examining associations between preterm birth and ADHD symptoms and diagnosis in adulthood, with evidence quality evaluation. We performed an Individual Participant Data (IPD) meta-analysis to determine if preterm and term-born adults differed in self-reported ADHD symptoms. In a population-based register-linkage study, we investigated the risk of receiving an ADHD diagnosis in preterm compared to term-born adults. We examined the effects of gestational age, birth weight, multiple birth status, bronchopulmonary dysplasia (BPD), and intraventricular hemorrhage (IVH) on later ADHD symptoms and diagnosis for preterm-born adults.

## Methods

The systematic review was performed in line with the preferred reporting items for Systematic Review and Meta-Analyses of Individual Participant Data (PRISMA IPD).^8^ The IPD meta-analysis and population-based register-linkage study were a part of the Research on European Children and Adults born Preterm (RECAP) consortium (https://recap-preterm.eu/) and Adults Born Preterm International Collaboration (APIC) (https://www.apic-preterm.org/members/). The analysis plan was submitted and approved by the RECAP consortium prior to the study (04/05/2019).

### Systematic search

**Fig. 1:**
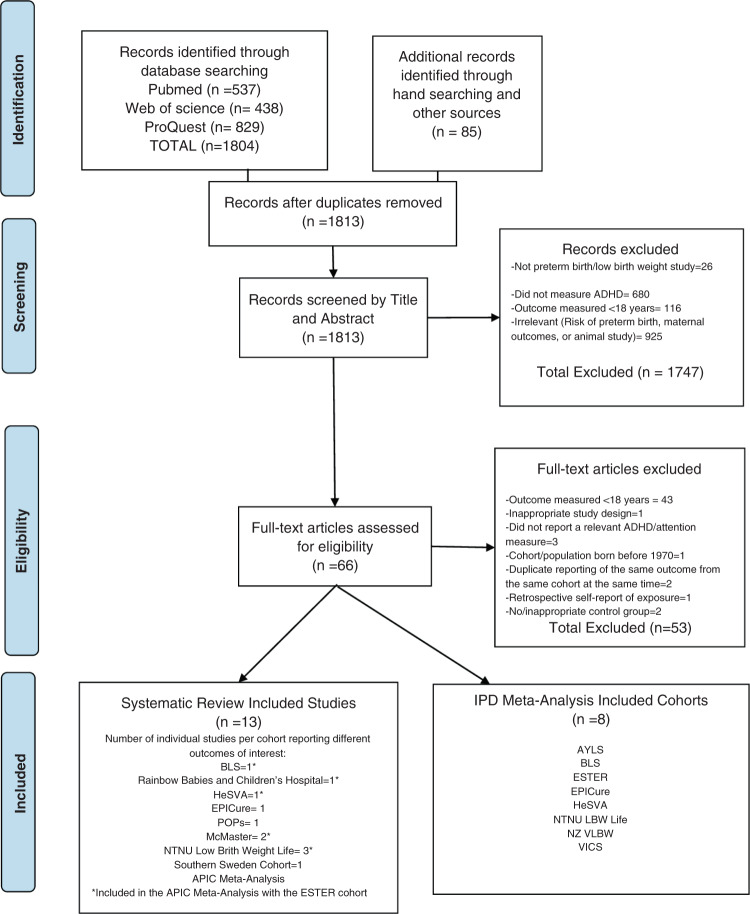
PRISMA flow diagram. Systematic search and selection of included studies.

We conducted systematic searches in PubMed, Web of Science, and PROQUEST from Inception to October 2019 and an updated search in April 2021 (Fig. 1 and Supplemental Table S1). Duplicate studies were removed, with remaining studies independently assessed in duplicate (RR, AL) first by title and abstract, followed by full-text assessment (Fig. 1). Additional studies were identified via the references of included studies and from relevant systematic reviews and meta-analyses.

### Eligibility criteria

Cohort, case–control, register-linkage studies, and meta-analyses assessing ADHD self-reported symptoms or diagnosis in adulthood (≥18 years) with data on gestational age, were included in the systematic review. Eligible symptom measures included a validated scale providing an overall ADHD symptom score, inattention, hyperactivity/impulsivity, attention problem scores, or clinical cutoffs. Eligible ADHD diagnosis included data from medical records, medical registers, or a structured diagnostic interview. Attention scales derived from neurocognitive tests were beyond the scope of this review. Due to profound changes in medicine during recent decades, we excluded studies with participants born before 1970. There were no language restrictions. Studies were excluded based on criteria presented in Supplemental Table S2.

Self-reported symptom studies, which met inclusion criteria for the systematic review and were a part of the RECAP project and APIC consortia were eligible to participate in the IPD meta-analysis. Cohorts with adult follow-up were invited to provide a data dictionary, which was checked for ADHD symptom scales. Studies that declined to provide a data dictionary, did not have an adult ADHD measure, or were not a part of the RECAP/APIC consortia were excluded.

In the population-based register-linkage study, the participants were identified from the Finnish Medical Birth Register. We included all live births in Finland from 01/01/1987 to 31/12/1998.

### Outcome measures

For the IPD, all included cohort studies utilized at least one ADHD self-reported symptom scale in adulthood (Supplemental Table S3). Primary outcomes in the IPD were total ADHD symptom *z*-score and probable clinical ADHD based on clinical cutoffs. Secondary outcomes were inattention, hyperactivity/impulsivity, and attention problems *z*-scores.

For the Finnish population-based register-linkage study, the primary outcome was primary or subsidiary ADHD diagnosis derived from the Care Register for Health Care with follow-up data at age 18 years or older available until 31/12/2016. This register carries all diagnoses registered during inpatient and outpatient visits in public specialized medical care classified using the International Statistical Classification of Diseases and Related Health Problems 10th (ICD-10) Revision (code: F90 for ADHD). The Care Register for Health Care is a validated research tool.^9^

### Exposure variables

Gestational age was calculated from the mother’s last menstrual period or by ultrasound. Preterm birth was further categorized into extremely preterm (EP; <28 + 0 gestational weeks + days), very preterm (VP; 28 + 0 to 31 + 6 gestational weeks + days), moderate-to-late preterm (MLP; 32 + 0 to 36 + 6 gestational weeks + days) and term (≥37 + 0 gestational weeks + days to 41 + 6 gestational weeks + days). Post term (>41 + 6 gestational weeks + days) births were excluded. Birthweight *z*-scores were determined using the Intergrowth 21 reference^10^ and small-for-gestational age (SGA) was defined as birthweight *z*-score < −2 SD and those not born SGA as birthweight −2 SD or above.

As cohort definitions varied, we harmonized BPD into “no BPD” versus “any BPD” and IVH as “no IVH” versus “any IVH” (Grades 1–4). In the register-linkage study, BPD was defined using the codes 770.7 (International Classification of Diseases 9th revision, ICD-9) and P27 (ICD-10) and IVH using codes 772.1 and 772.2 (ICD-9) and P52 (ICD-10). In both studies, multiple births were classified as a binary variable (0 = singleton, 1= multiple).

### Covariates

Covariates included participant’s sex, age (at follow-up[cohorts]/at emigration, death or end of follow-up [31/12/2016] [register-linkage study]), and parental education (of either parent/of the mother), which was harmonized according to the International Standard Classification of Education (ISCED) into low (ISCED level 0–2), medium (3–5), and high (6–8).^11^ Neurosensory impairment (NSI) was accounted for in sensitivity analyses. For the cohorts, NSI was defined as “no NSI” versus “any NSI” if the participant had severe visual (blind in both eyes) or hearing impairment (not corrected by hearing aids), cerebral palsy, or cognitive impairment (childhood IQ < 70). Missing NSI data in cohorts were treated as no evidence of NSI. In the register study, NSI was defined with the codes 369, 389, 343, and 318–319 (ICD-9) and F70-F79, H54, H90, H91, and G80 (ICD-10).

### Quality of evidence assessment

Two reviewers (RR, AL) independently assessed the evidence quality of studies included in the systematic review using the Newcastle–Ottawa–Scale (NOS).^12^ The assessment criteria for each NOS domain (Selection, Comparability, Outcome) were set out at the onset of the study (Supplemental Table S4). Assessment disagreements between the two reviewers (RR, AL) were resolved based on consensus with a third reviewer (ML-P, KH).

### Data sources

IPD and data dictionaries were provided by each of the participating cohorts via the RECAP platform data nodes. The adult data were cleaned and harmonized by the University of Helsinki (RR and reviewed by PG). Perinatal data for the IPD were harmonized by the University of Warwick. The register data were cleaned and harmonized by the Finnish Institute for Health and Welfare. The data underlying this article cannot be shared publicly due to the personal and sensitive information of the participants. If requested, access to this data is subject to data sharing agreements.

### Statistical analysis

The IPD meta-analysis was conducted using a one-stage meta-analytic approach where IPD from all cohort studies were analyzed simultaneously. We applied linear mixed-effects models with the restricted maximum likelihood estimation method for the covariance parameters to examine mean differences with 95% confidence intervals (95% CI) in ADHD symptoms between preterm and term-born adults. We applied generalized linear mixed-effects models for binomial data to estimate the odds ratio (OR) with 95% CI of scoring above the clinical cutoff on any of the ADHD scales in preterm compared with term-born adults. In mixed-effects models, random intercepts were specified to account for the clustering of participants within cohorts.

ADHD scores were normalized with rank normalization and standardized to the mean of 0 and SD of 1 within each of the studies. As all but two (VICS, NZ VLBW) of the included cohorts had two different ADHD measures, the mean of available standardized ADHD scores was taken.

In the register study, we applied binomial logistic regression to estimate the relative risk (RR) with 95% CI of being registered with ADHD diagnosis in public specialized medical care in adulthood between preterm compared with term-born adults.

In both study designs, we tested whether the associations varied according to the degree of prematurity and SGA status. Then we tested whether gestational age, birth weight *z*-score, multiple births, BPD, and IVH were associated with ADHD symptoms and diagnosis among adults born preterm. The effect sizes were estimated in adjusted models. Finally, in sensitivity analyses, we tested whether excluding participants with NSI affected the associations and in the register study, we also excluded participants who died or emigrated at 18 years of age or older.

## Results

### Systematic review

Eleven individual studies and one meta-analysis met our inclusion criteria, representing 9 clinical cohorts examining 12 adult self-reported outcomes and two diagnostic interviews (Table 1). Overall, included studies were mostly moderate to high methodological quality (Table 1 and Supplemental Table S4) with scores ranging from 3 to 8 out of 9 possible points. Regarding Selection, five cohorts focused on VP/VLBW, while three cohorts focused on EP/ELBW participants. None of the studies received all possible points, particularly due to the lack of adjustment for familial confounding either via parental ADHD or sibling comparisons. However, eight of the cohorts adjusted their analysis for at least three of the following factors, including participant age, sex, maternal age at birth, maternal/parental education, and family socioeconomic status. Regarding the Outcome, only two studies^3,13^ had more than 50% attrition rate. Neither of the two cohorts (NTNU LBW Life, McMaster) which utilized diagnostic interviews (MINI), observed differences between VLBW/ELBW preterms and normal birth weight (NBW) term controls.^14,15^ Of the 11 studies,^2,3,13,16–21^ with adult self-reported questionnaires, only three cohorts (BLS, EPICure, NTNU LBW Life) found differences between preterms and term controls (Table 1). As all but two of the studies^3,21^ were previously meta-analyzed,^22^ and no difference was observed, an aggregate data meta-analysis was not duplicated here.

**Table 1:** Summary of the findings and quality of evidence assessment using the Newcastle–Ottawa–Scale of the studies included in the systematic review comparing adults (≥18 years) born preterm and at term or with normal birth weight in self-reported symptoms of attention-deficit/hyperactivity disorder

Cohort	Country	Birth year	*N*	Group	GA, wk	BWT, g	Age, y	Measure	Results	Citation	Quality of evidence			
											Selection	Comparability	Outcome	Quality of evidence
*BLS^a^	Southern Bavaria, Germany	1985–1986	260	VP^m^/VLBW^o^	≤32	<1500	22–26	ASEBA^s^ YASR^r^	*F*(1,420) = 39.45^††^	Breeman et al.^2^	3	1	2	6
			229	Term	≥37	≥2500		ARS-DSM IV^t^	RR 3.29, (95% CI 1.39–7.81)^†^					
Cleveland^b^	Cleveland, Ohio, USA	1977–1979	241	VLBW^o^	29.7	<1500	20	ASEBA^s^ YASR^r^	**MD (95% CI)**	Hack et al.^16^	2	0	2	4
									Men −0.2 (−0.9, 0.4)					
									Women -0.1 (−0.7, 0.5)					
			232	NBW^p^ Term**	≥37	≥2500		ARS-DSM IV^t^	***M*** **(SD)**					
									VLBW men 4.6 ± 4					
									NBW men 4.8 ± 3					
									VLBW women 4.2 ± 4					
									NBW women 3.8 ± 3					
*EPICure^c^	UK & the Republic of Ireland	1995	117	EP^l^	<28		19	SDQ^w^	**Mean difference**	Linsell et al.^3^	2	0	1	3
									HI/IA MD 1.8 (1.0–2.7)^†^					
			55	Term*	≥37				Sig. difficulties RR 3.80 (2.52, 5.74)^†^					
*HeSVA^d^	Helsinki, Finland	1978–1985	162	VLBW^o^	24–36	<1500	18–27	APQ^u^	***M*** **(SD)**	Strang-Karlsson et al.^9^	4	1	2	7
			172	TERM	≥37									
									Total problem scores					
McMaster^e^	Ontario, Canada	1977–1982	142	ELBW		<1000	22–26; 29–36	ASEBA^s^ YASR^r^	***M*** **(SD)**	Boyle et al.^13^	2	1	2	5
									ELBW 2.43(2.13)					
									NBW 2.09 (1.95)					
			133	NBW^p^**		≥2500		MINI^v^	**OR (95% CI)**	Van Lieshout et al.^14^	2	1	2	5
									Current disorder inattentive subtype 7.37 (0.80, 68.11)					
*NTNU LBW Life^f^	Trondheim, Norway	1986–1988	44	VLBW^o^	<37	≤1500	26	MINI^v^	***n*** **(%)**	Laerum et al.^15^	4	1	3	8
									Preterm VLBW 4 (9)					
									Term 1(1)					
			81	Term	≥37	≥10th percentile		ASEBA ASR^q^	***M*** **(SD)**	Laerum et al.^20^	4	1	2	8
									Attention problems					
									Preterm VLBW 7.0 (4.8)					
									Term 4.6 (4.3)^†^					
							20	ARS-DSM IV^t^	Participants report no differences	Lund et al.^17^	4	1	2	7
*NZ VLBW^g^	New Zealand	1986	230	VLBW^o^		<1500	22–23	ARS-DSM IV^t^	**MD (95% CI)**	Darlow et al.^18^	2	1	2	5
			69	Term**	≥37				0.6 (−1.1, 2.3)					
Southern Sweden Cohort^h^	Sweden	1985–86	52	EP^l^	<29	1002(234)	18.4 (.2)	ASEBA YASR^r^	***M*** **(SD)**	Hallin et al.^21^	4	0	2	6
									EPT 4.0 (2.7)					
			54	Term**	≥37				Terms 4.0 (2.3)					
**Meta-analyses on adult ADHD symptoms** (Note: The meta-analyses include some of the empirical studies listed above. Meta-analyses that did not meta-analyze adult data are not included, nor studies without any self-reported ADHD symptoms).														
APIC^j^	Finland^e,i,k^, USA^d^, Canada^f^, Norway^g^, Germany^c^	1977–1989	747	Preterm VLBW^o^ ELBW^n^	<37	<1500; <1000	18–27	ASEBA^s^ ASR^q^ YASR^r^	**Beta (95% CI)**	Pyhälä et al.^22^				*NA*
									Attention problems 0.04 (−0.02, 0.11)					
									ADHD total score −0.02 (−0.10, 0.06)					
			1512	Term	≥37	≥2500								
									*I*^2^ = 57.5%, *p* = 0.038					

Each study has its own Quality of Evidence rating as relevant to our study question; therefore, different studies from the same cohort may have different QoE ratings, or may even receive a different rating based on a different study question. As the relevant individual studies have been assessed above, the QoE of the meta-analyses was not assessed.

*Cohort provided data for the IPD meta-analysis.

**Controls recruited later from a different source.

^†^*p* ≤ 0.05.

^††^*p* ≤ 0.001.

^a^Bavarian Longitudinal Study.

^b^Rainbow Babies and Children’s Hospital of Cleveland.

^c^EPICure 1995.

^d^Helsinki Study of very low birth weight adults.

^e^McMaster University.

^f^University of Trondheim, Norway Low Birth Weight Life Cohort.

^g^New Zealand Very Low Birth Weight 1986 Cohort.

^h^Southern Sweden Cohort.

^i^[Preterm Birth and Early Life Programming of Adult Health and Disease] Ennenaikainen syntymä, raskaus ja lapsen terveys aikuisiässä (ESTER) Cohort.

^j^Adults Born Preterm International Collaboration (APIC).

^k^Arvo Ylppö Longitudinal Study (AYLS).

^l^Extremely preterm.

^m^Very preterm.

^n^Extremely low birth weight.

^o^Very low birth weight.

^p^xxxx.

^p^Normal birth weight.

^q^Achenbach system of empirically based assessment—adult self report.

^r^Achenbach system of empirically based assessment—young adult self report.

^s^Achenbach system of empirically based.

^t^ADHD Rating Scale DSM IV.

^u^Adult problems questionnaire.

^v^Mini-international neuropsychiatric interview.

^w^Strengths and difficulties questionnaire.

### IPD meta-analysis

Eight^19,23–27^ out of 11 RECAP/APIC cohorts with ADHD symptoms reported in adulthood provided IPD for 1385 preterm and 1513 term borns (Table 2). The cohorts came from Finland (*n* = 3; AYLS, HeSVA, Ester),^23,24,26^ Germany (*n* = 1; BLS),^28^ UK & Ireland (*n* = 1, EPICure),^25^ Norway (*n* = 1, NTNU LBW Life),^27^ Australia (*n* = 1, VICS)^29^ and New Zealand (*n* = 1, NZ VLBW).^18^ The POPs cohort (The Netherlands, VPT, *n* = 1338)^30^ provided data but was subsequently excluded for lack of a control group. The two North American cohorts identified that did not provide data were the McMaster (Canada, ELBW, *n* = 175)^13^ and the Rainbow Babies and Children’s Hospital (USA, VLBW; *n* = 473).^16^ For all three RECAP/APIC studies not included in the IPD meta-analysis,^13,16,30^ the previous findings are indicated in Table 1.

**Table 2:** Characteristics of the cohort studies included in the Individual Participant Data meta-analysis and population-based register-linkage study according to preterm and term birth

Characteristics	Preterm (<37 + 0 wks + days)		Term-born controls (37 + 0–41 + 6 wks + days)	
	*N* or *M*	% or SD	*N* or *M*	% or SD
Participants, *n*				
All Cohorts	1385	45.9	1633	54.1
AYLS^a^	171	19.0	727	81.0
BLS^b^	234	51.5	220	48.5
EPICure^c^	117	69.6	51	30.4
ESTER^d^	372	54.1	315	45.9
HeSVA^e^	111	53.4	97	46.6
NTNU^f^	60	42.3	82	57.7
NZVLBW^g^	230	76.9	69	23.1
VICS^h^	90	55.6	72	44.4
Population-based register-linkage study^i^	37,538	5.2	691,616	95.9
Sex, female				
All Cohorts	721	52.1	891	55.3
AYLS	85	49.7	397	54.6
BLS	109	48.2	117	51.8
EPICure	64	54.7	32	62.7
ESTER	201	54.2	170	54.0
HeSVA	62	55.9	55	56.7
NTNU	28	46.7	48	58.5
NZVLBW	126	54.8	36	52.2
VICS	46	51.1	36	50.0
Population-based register-linkage study	17,082	45.5	339,994	
Gestational age, wk				
All Cohorts	30.9	3.9	39.6	1.3
AYLS	33.9	2.6	39.4	1.3
BLS	30.5	2.1	39.6	1.1
EPICure	24.5	0.7	–	–
ESTER	34.3	2.3	39.9	1.2
HeSVA	29.3	2.4	40.0	1.1
NTNU	29.1	2.7	39.6	1.0
NZVLBW	29.2	2.5	–	–
VICS	27.0	2.4	39.0	1.2
Population-based register-linkage study	34.3	2.3	39.6	1.2
Birthweight *z*-score				
All Cohorts	−0.02	1.2	0.61	1.1
AYLS	0.42	1.1	0.74	1.1
BLS	−0.45	1.4	0.21	0.985
EPICure	−0.55	0.736	–	–
ESTER	0.19	1.1	0.57	1.0
HeSVA	−0.44	1.2	0.63	1.1
NTNU	0.06	1.1	0.97	0.9
NZVLBW	−0.32	1.2	–	–
VICS	0.07	1.2	0.37	0.9
Population-based register-linkage study	0.17	1.1	0.68	1.0
Small-for-gestational age (<−2 SD), yes				
All Cohorts	55	4.0	*	*
AYLS	*	*	13	1.8
BLS	12	5.1	7	3.2
EPICure	*	*	–	–
ESTER	15	4.0	11	3.5
HeSVA	8	7.2	3	3.1
NTNU	*	*	0	0
NZVLBW	13	5.7	–	–
VICS	*	*	*	*
Population-based register-linkage study	1227	3.3	4812	0.7
Age at follow-up, years^j^				
All Cohorts	23.6	2.5	24.6	2.1
AYLS	25.5	0.6	25.5	0.6
BLS	26.3	0.7	26.3	0.7
EPICure	19.3	0.6	19.2	0.6
ESTER	23.1	1.3	23.4	1.2
HeSVA	24.6	2.1	24.6	2.2
NTNU	26.4	0.6	26.5	0.5
NZVLBW	23.4	0.5	23.7	0.6
VICS	18.6	0.5	18.7	0.5
Population-based register-linkage study	34.31	2.34	39.59	1.22
Highest parental education attained^k^, lower secondary or less				
All Cohorts	198	14.3	145	8.9
AYLS	16	9.5	75	10.4
BLS	29	12.7	31	15.1
EPICure	22	19.8	2	3.9
ESTER	32	8.7	19	6.07
HeSVA	11	10.1	6	6.2
NTNU	8	11.9	4	4.6
NZVLBW	65	29.0	–	–
VICS	15	29.8	8	27.6
Population-based register-linkage study	4776	12.7	69,768	10.1
Neurosensory impairment, any				
All Cohorts	121	8.7	27	1.7
AYLS	16	13.2	26	3.6
BLS	44	36.4	1	0.4
EPICure	22	18.2	–	–
ESTER	7	5.8	–	–
HeSVA	5	4.1	–	–
NTNU	6	5.0	–	–
NZVLBW	21	17.4	–	–
VICS	0	0	–	–
Population-based register-linkage study	1774	4.7	8383	1.2
Preterm only characteristics				
*Bronchopulmonary dysplasia*				
All Cohorts	323	38.6	–	–
AYLS	*	*	–	–
BLS	124	38.4	–	–
EPICure	84	26.0	–	–
ESTER	–	–	–	–
HeSVA	25	7.7	–	–
NTNU	13	4.0	–	–
NZVLBW	48	14.9	–	–
VICS	28	8.7	–	–
Population-based register-linkage study	587	1.6	–	–
*Intravehicular hemorrhage. Grades 1–4*				
All Cohorts	246	25.7	–	–
AYLS	7	2.8	–	–
BLS	46	18.7	–	–
EPICure	78	31.7	–	–
ESTER	–	–	–	–
HeSVA	14	5.7	–	–
NTNU	6	2.4	–	–
NZVLBW	62	25.2	–	–
VICS	33	13.4	–	–
Population-based register-linkage study	148	0.4	–	–

*Cohort counts between 1 and 5, and where only one cohort has less than five the overall total of the cohorts are not presented to protect the privacy of the participants.

– Data not available.

^a^Arvo Ylppö Longitudinal Study (Uusimaa, Finland, born 1985).

^b^Bavarian Longitudinal Study, also known as BEST (Bayerische Entwicklungsstudie) (Germany, born 1985).

^c^EPICure Cohort (UK & The Republic of Ireland, born 1995).

^d^[Preterm Birth and Early Life Programming of Adult Health and Disease] Ennenaikainen syntymä, raskaus ja lapsen terveys aikuisiässä, Northern Finland, born 1985–1989).

^e^Helsinki Study of Very Low Birth Weight Adults (Helsinki, Finland, born 1978–1985).

^f^*Norges Teknisk-Naturvitenskapelige Universitet* [Norwegian University of Science and Technology] Low Birth Weight Life (Trondheim, Norway, born 1986–1988).

^g^New Zealand Very Low Birth Weight 1986 Cohort (New Zealand, born 1986).

^h^Victorian Infant Collaborative Study (Victoria, Australia, born 1991–1992).

^i^All live births in Finland between 01/01/1987 and 31/12/1998.

^j^For the population-based register-linkage study age is at death, emigration or end of follow-up (31/12/2016).

^k^For the population-based register-linkage study highest education is maternal education.

All IPD data sets were checked for data integrity (RR, PG). Any questions were resolved with representatives of specific cohorts. Ultimately, no major issues were identified with the data sets.

Characteristics of the cohorts included in the IPD meta-analysis are in Table 2. Raw cohort-specific mean values of ADHD total and subscale scores for preterm and term-born controls are shown in Supplemental Table S3 and the number of participants within each cohort and across the different cohorts contributing to the IPD meta-analysis according to the degree of preterm birth are in Supplemental Table S5. Of the covariates, lower parental education, and female sex were associated with higher total ADHD symptoms *z*-score and probability to score above the ADHD clinical cutoff (Supplemental Table S6).

**Fig. 2:**
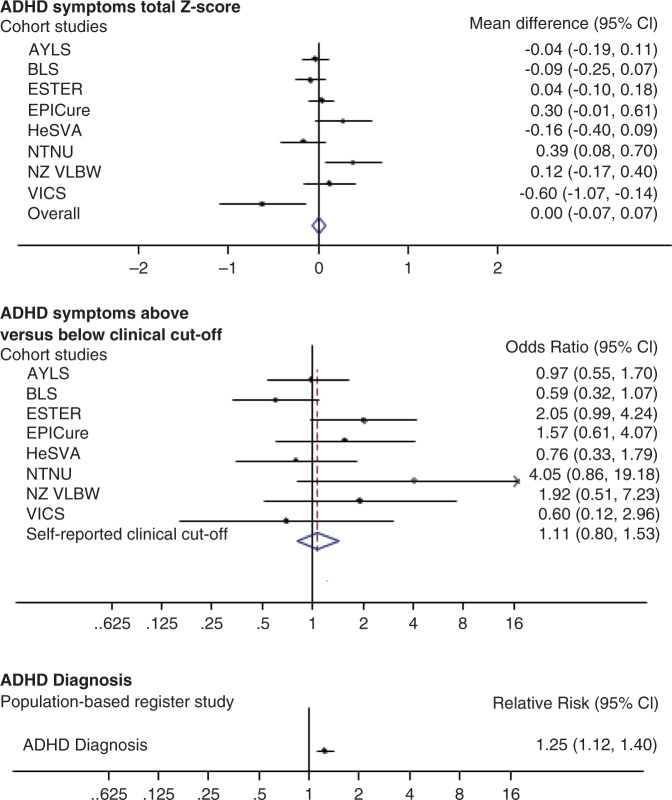
ADHD symptoms and diagnosis by Cohort. Mean differences between preterm (<37 weeks) and term-born (37 + 0–41 + 6 wks + days) adults (≥18 years) in self-reported attention-deficit hyperactivity disorder (ADHD) symptoms z-scores and in the odds to score above versus below the ADHD clinical cutoffs in the Individual Participant Data meta-analysis of cohort studies, and in the relative risk to receive ADHD diagnosis in adulthood in the population-based register-linkage study. Error bars refer to 95% confidence intervals.

Preterm birth was not associated with total ADHD symptoms *z*-score, symptoms above the clinical cutoff (Fig. 2), hyperactivity-impulsivity, inattention, or attention problems *z*-scores (Supplemental Fig. 1). We individually compared EP, VP, MLP, SGA preterms, and preterms not born SGA groups with term controls (Supplemental Table S7). There were no differences, except adults born EP had higher odds of scoring above the ADHD clinical cutoff. However, EP and term controls showed no differences when we excluded participants with NSI (*n* = 148) (*p* = 0.18; data not shown).

In preterm only analyses, gestational age, birth weight *z*-score, multiple birth, and BPD were not associated with ADHD symptoms in adulthood (Table 3). ADHD symptoms *z*-scores were higher for preterm borns with IVH than with no IVH (Table 3). This difference remained when we excluded participants with NSI (*p* = 0.008; data not shown).

**Table 3:** Associations between gestational age, birth weight *z*-score, multiple births, and neonatal complications with self-reported attention-deficit/hyperactivity disorder (ADHD) symptoms in adulthood in the individual participant data meta-analysis of cohort studies and with ADHD diagnosis in adulthood in the population-based register-linkage study among those born preterm.

Predictors	Individual participant data meta-analysis (*n* = 1385)								Population-based register-linkage study (*n* = 37,537)			
	ADHD symptoms *z*-score				ADHD symptoms above versus below clinical cutoff				ADHD diagnosis yes versus no			
	Estimate	95% CI		*p*	OR	95% CI		*p*	RR	95% CI		*p*
Gestational age (weeks)	0.00	−0.01	0.01	0.73	0.95	0.90	1.00	0.07	0.93	0.89	0.97	<0.001
Birth weight *z-*score (standard deviation units)	0.01	−0.03	0.06	0.59	1.02	0.89	1.18	0.76	0.88	0.80	0.97	<0.001
Multiple birth												
Singleton	Ref				Ref				Ref			
Multiple	−0.11	−0.24	0.01	0.08	0.83	0.56	1.24	0.22	0.83	0.62	1.11	0.20
Bronchopulmonary dysplasia												
No	Ref				Ref				Ref			
Yes	0.05	−0.09	0.19	0.47	1.14	0.73	1.79	0.57	1.83	0.95	3.53	0.07
Intraventricular hemorrhage												
No	Ref				Ref				Ref			
Yes	0.17	0.02	0.32	0.03	1.53	0.97	2.44	0.07	0.84	0.12	5.96	0.86

Estimate refers to mean difference in ADHD symptoms between categorical predictors and standard deviation unit change per each unit change in the predictor. All associations are adjusted for participant’s sex, age (in adulthood follow-up in the meta-analysis of cohorts and age at death, emigration or end of follow-up [31/12/2016] in the register-linkage study), and parental education (of either parent in the meta-analysis of cohorts and of maternal in the register-linkage study).

*OR* odds ratio, *RR* relative risk, *95% CI* 95% confidence interval.

### Population-based register-linkage study: ADHD diagnosis

The register study characteristics are described in Table 2. Supplemental Table S5 provides the number of participants according to the degree of preterm birth. Of the entire population of 729,154 individuals, 4683 (0.64%; 308 of 37,538 [0.82%] preterm, 4375 of 691,616 [0.63%] term) were registered with an ADHD diagnosis while attending specialty care at age 18 years or older. Of the covariates, lower maternal education, male sex, and older age were associated with higher RR for ADHD diagnosis in adulthood (Supplemental Table S6).

Compared to term-born controls, the RR of ADHD diagnosis in adulthood was higher for preterms born SGA and not SGA and for those born VP (Supplemental Table S7).

In the preterm-only analyses, the RR for ADHD diagnosis decreased according to each increasing week of gestation and SD in birth weight *z*-score (Table 3). Multiple births, BPD, or IVH were not associated with the risk of ADHD diagnosis in adulthood (Table 3).

The associations remained when we excluded those individuals who died (*n* = 25) or emigrated (*n* = 90) after 18 years of age and those with NSI (*n* = 10157) (*p*-values < 0.01; data not shown). After excluding individuals with NSI, two associations became apparent: RR of ADHD diagnosis was higher for EP than term-born controls (RR = 2.06, 95% CI 1.08, 3.94, *p* = 0.03) and for preterms with any BPD compared with those with no BPD (RR = 2.52, 95% CI 1.26, 5.05, *p* = 0.01).

## Discussion

This systematic review highlighted the conflicting evidence of previous studies that varied in methodological quality from moderate to high. In the IPD meta-analysis the level of self-report ADHD symptoms in adulthood was similar in preterm and term controls. In contrast, the population-based register-linkage study revealed that adults born preterm, EP, VP, and preterms born SGA and preterms not born SGA had a higher RR for being registered with ADHD diagnosis in public specialized medical care in adulthood compared to term controls. Moreover, among preterms in the register-linkage study, the RR of ADHD diagnosis in adulthood decreased according to each weekly increase in gestation and SD increase in birth weight *z*-score. The associations in the IPD meta-analysis or the register-linkage study were not explained by participant’s sex, age, and parental/maternal education. Moreover, the associations changed only a little when we excluded participants with NSI.

The conflicting information of the previous studies identified by the systematic review reflects methodological differences between the studies and limitations that relate to small sample sizes and different control group recruitment approaches. Several studies also lack standardized adjustments for covariates, gestational age group assessments, and adult-specific follow-ups. We were able to address some of the limitations of the individual cohort studies in the IPD meta-analysis and the register-linkage study.

The discrepant IPD meta-analysis and the register-linkage study findings may relate to differences in the severity of ADHD symptoms detected by the meta-analysis of self-reports and register-linkage study of diagnosis. ADHD self-reports may have captured milder, sub-clinical symptoms, whereas the register-linkage study on ADHD diagnosis may have captured the most severe end of the ADHD spectrum, attending public specialized medical care. Only 0.64% of the individuals in our study had been registered with an ADHD diagnosis in adulthood. This is slightly lower than the estimated adult prevalence of 2.5–4.2% in high-income countries.^6^ Hence, the specialized medical care diagnosis may miss a broader spectrum of ADHD cases. Furthermore, we cannot preclude informant bias, as self-reports are subject to response-bias. Reported simultaneously, adult self-reports and parent reports of the same participants frequently vary.^2^ In order to receive an ADHD diagnosis in adulthood, information is gathered from multiple informants, most importantly from the individual themself, but also from partners, parents, siblings, or school records. Then a physician makes the diagnosis according to the specific diagnostic criteria for ADHD. For the IPD meta-analysis, we cannot rule out selective data attrition in the cohorts, as adults with more severe ADHD symptoms may have been more likely to drop out, eliminating the possible detection of associations. In the register-linkage study, data attrition was minimal. While this precludes bias of selective drop-out, we cannot rule out that preterms may have been more likely to be registered with ADHD diagnosis in adulthood, if they have visited health care more frequently for other preterm-related conditions. Although the IPD meta-analysis (Cohen *d* = 0.10, OR = 1.34) and the register-linkage studies (RR = 1.20) provided ample statistical power (80% at alpha = 0.05) to detect small effect sizes, the statistical power in the register-linkage study was superior. This is particularly true for comparisons of ADHD symptoms above the clinical cutoff in the IPD meta-analysis and ADHD diagnosis in the register-linkage study. The statistical power is, however, smaller in comparisons of different gestational age groups and in the comparisons limited to preterm borns only. Moreover, the discrepancies may also be attributable to the composition of the total IPD sample which comprised preterm and term-born individuals from seven different countries, while the register-linkage study comprised only Finns. It is also important to note, that due to differences in the study designs, the samples included in the IPD meta-analysis comprised different numbers of different gestational age groups. For instance, 36.7% of the total IPD EP participants came from the UK, 20.7% from Australia, 17.9% from New Zealand, 13.7% from Finland, 5.6% from Norway, and 5% from Germany., whereas over 70% of MLP and term-born gestational age groups came from Finland. That preterm and term-born adults self-reported equal levels of ADHD symptoms may also suggest that ADHD symptoms do not hinder the daily functioning of adults born preterm. While ADHD in the general population is associated with risk-taking behaviors and criminality,^31–33^ preterm-born individuals report less externalizing behaviors,^22^ less smoking and substance abuse,^19,34^ fewer contacts with police,^34^ have less often experienced sexual intercourse^35,36^ by young adulthood, and they also less often have substance use disorders and criminal convictions.^4^ The lack of differences may also reflect preterm personality: in comparison to term borns preterm borns report being more conscientious, shyer, and less impulsive, less excitement seeking, and less open to new experiences.^37,38^

The lack of differences between preterm and term adults in self-reported symptomatology in the IPD meta-analysis are consistent with a previous meta-analysis,^22^ which however included fewer cohorts with adult follow-up than included here. The register-linkage study findings indicating higher RR for ADHD diagnosis for preterms than term controls are consistent with another Finnish register study, which examined ADHD specialty care diagnosis between ages 3 and 19,^1^ and with a Swedish register study assessing specialty care diagnoses at ages 5–19 y.^4^ Our findings are also in agreement with Norwegian, and one Swedish, register studies assessing psychostimulant medication use at 18–38 y,^39^ 30 y,^40^ and at 26–33 y.^41^

Highlighting further differences between the results of the IPD meta-analysis and register-linkage study, women in the IPD meta-analysis of cohorts reported higher levels ADHD symptoms, while in the register-linkage study men had a higher RR for ADHD diagnosis in adulthood. The higher ratio of men to women, may suggest that women may be more likely to be underdiagnosed.^6^ This may be due to women with ADHD, at least in childhood, presenting less disruptive ADHD-like behaviors,^42^ and in parent and teacher reports girls with ADHD show lower levels of hyperactivity, impulsivity, inattention and externalizing symptoms and higher levels of internalizing symptoms.^32^ However, it has also been suggested that differences between men and women in ADHD may level off as the individuals’ age, and some studies have reported that in adulthood women report higher ADHD symptomatology than men.^6,43^

The only agreed finding between the cohort IPD meta-analysis and the register-linkage study was that participants with lower parental/maternal education had higher ADHD symptoms and RR for diagnosis.

A strength of our systematic review and IPD meta-analysis includes a focused age group, allowing for a better understanding of the symptomatology of adults, instead of summarizing the symptoms or diagnosis across the entire lifespan as done in other studies.^44^ This is important, as generally, the rates of ADHD tend to decline with age.^5^ Additionally, the limited range of birth years included allows contextualization of the medical practices or interventions, which may have played a role in the outcomes observed in this generation. Furthermore, performing an IPD meta-analysis allows for the same covariates and confounders to be accounted for across all studies, typically a weakness in standard aggregate data meta-analyses. Moreover, in both the IPD meta-analysis and the register-linkage study, we were able to uniquely parcel out gestational age and birth weight categories and study neonatal complications in relation to the risk of ADHD symptoms and diagnosis in those born preterms, for which individual cohort studies lack the power to do.

A common weakness, our results do not represent low- or middle-income countries, as the IPD meta-analysis included only high-income country populations.^44^ Hence, the generalizability of the findings is limited. Additionally, although we had IPD data, we were not able to account for additional potential confounders, and residual confounding cannot be excluded. We were not able to obtain IPD from all cohorts with self-reported ADHD symptom scales in adulthood with preterm or VLBW participants. It is unlikely that the inclusion of these three studies would have changed the results, since these studies have previously reported no differences.^13,16,21^ Finally, while the Care Register for Health Care is a validated research tool, including ADHD diagnosis in childhood,^9^ comparable data on adulthood ADHD diagnosis is lacking.

Considering the substantial economic burdens associated with preterm birth^45^ and with ADHD,^46^ understanding the intersections of the two is important. Pinpointing that preterms or specific preterm and birth weight groups are at a slightly increased risk of receiving ADHD diagnosis in adulthood than others may pave the way for targeted interventions to reduce the burden. On the other hand, the lack of differences in self-reported ADHD symptoms between preterm and term-born adults delivers a positive message for the preterm-born individuals themselves, their families, and healthcare professionals.

## Supplementary information

Supplementary information

Supplementary Material
