# Supplementary material for: ADHD symptoms and diagnosis in adult preterms: systematic review, IPD meta-analysis, and register-linkage study
Source: Pediatr Res. 2022 Jan 7;93(5):1399–409. doi: 10.1038/s41390-021-01929-1 (PMC10132969; doi:10.1038/s41390-021-01929-1)
Supplement: Supplementary file 2 — Supplementary Material [file 41390_2021_1929_MOESM2_ESM.pdf]

Supplementary Figure 1

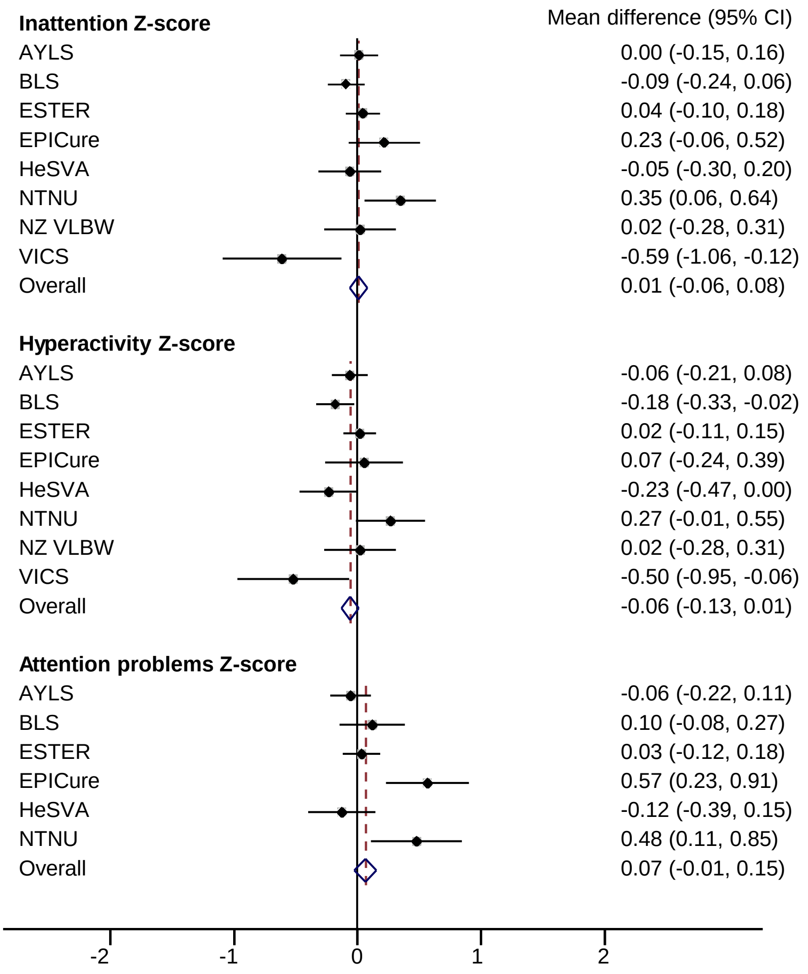

Supplemental Table S1. Search strategies to identify studies that have compared adults (≥18 years) born preterm and at term or with normal birth weight in attention deficit / hyperactivity disorder (ADHD) symptoms self-reports or in ADHD diagnosis**.**

**Database**	**Strategy**
Search performed in PubMed<br>on 31-10-2019<br>Yielded 537 results.	(((((((((((((((((((Neuro* OR neuropsych* OR neurodevel*)) OR (ADHD OR 'attention deficit hyperactivity disorder' OR attention deficit/hyperactivity disorder')) OR (autism OR autistic OR 'autism spectrum disorders')) OR (Mood OR bipolar OR depress*)) OR (anxiety OR obsessive-compulsive)) OR (disruptive OR externalizing OR internalizing OR impulsiv* OR behavior OR conduct)) OR (attachment OR PTSD OR adjustment)) OR (pica OR rumination OR anorexia OR bulimia OR binge-eating disorders OR 'eating disorder*)) OR (schizophrenia OR schizotypal personality OR schizoaffective OR psych* OR pyschological OR pyschiartic OR personality) OR (elimination OR enuresis OR oncopresis)) OR (somatic OR 'illness anxiety conversion' OR conversion)) OR (substance related OR substance-related OR smok* OR alcohol* OR gambling)) OR ('sleep disturbance*' OR 'circadian rhythm' OR hypersomnolence OR narcolep* OR parasomnias OR 'sleep apnea')) AND (symptoms OR disorders)) AND (very preterm OR preterm OR <32 weeks OR less than 32 weeks OR 32 week* OR ELBW OR VPT OR gestational age OR)) AND (adulthood OR adult Or long-term OR long term)) AND english[Language])) NOT (animal OR rat*))
Searched performed in Web of Science on 1-11-2019.<br>Yeilded 438 Results. //	**TOPIC:** (Preterm born adults OR ELBW OR VPT OR extremely low birth weight OR SGA) *AND* **TOPIC:** (mental health OR behavior OR psych* Or depress* OR ADHD OR anx* OR attention OR schizo* OR personalit* OR internal* OR external* OR conversion OR substance* OR sleep OR alcohol OR impulsiv* OR bipolar OR mood OR obsessive OR attachment OR conduct OR disruptive) *AND* **TOPIC:** (symptom* OR disorder*) *NOT* **TOPIC:** (rat* OR mice* OR rodent* OR rabbit) *NOT* **TOPIC:** (maternal)<br>*Indexes=SCI-EXPANDED, SSCI, A&HCI, CPCI-S, CPCI-SSH, BKCI-S, BKCI-SSH, ESCI, CCR-EXPANDED, IC Timespan=All years*<br><br>(neuro* OR ADHD OR Attention deficit hyperactivty disorder OR autis* OR mood OR bipolar OR depress* OR anxiety OR anxious OR obsessive-compulsive OR disruptive OR externali* OR internali* OR impulsiv* OR behavior OR attachment OR conduct OR schizophrenia OR schizo* OR psych* OR personality OR elminination OR enuresis OR oncopresis OR somatic OR conversion OR substance* OR smok* OR alcohol* OR gambl* OR sleep distrubance OR circadian rhythm OR hyperomnolence OR narcolep* OR parasomnias OR sleep apnea) AND TOPIC:(symptom* OR disorder*) AND TOPIC: (preterm OR very preterm OR VPT OR gestational age OR ELBW) AND TOPIC: (adult*) NOT TOPIC: (animal OR rat) Indexes=SCI-EXPANDED, SSCI, A&HCI, CPCI-S, CPCI-SSH, BKCI-S, BKCI-SSH, ESCI, CCR-EXPANDED, IC
PROQUEST Search<br>04-11-2019/ yielded 838	((adult*) AND (mental health OR behavior OR psych* OR depress* OR ADHD OR anx* OR attention OR schizo* OR personalit* OR internal* OR external* OR conversion OR substance* OR sleep OR alcohol OR impulsiv* OR bipolar OR mood OR obsessive OR attachment OR conduct OR disruptive) AND (symptom* OR disorder*) NOT (rat OR mouse OR mice OR rabbit OR rodent) NOT diabetes NOT (pharma* OR medication OR intervention)) AND stype.exact("Conference Papers & Proceedings" OR "Encyclopedias & Reference Works" OR "Books" OR "Working Papers" OR "Scholarly Journals" OR "Dissertations & Theses" OR "Evidence-Based Medical Resources") AND at.exact("Book Chapter" OR "Research Topic" OR "Letter To The Editor" OR "Annual Report" OR "Dissertation/Thesis" OR "Government & Official Document" OR "Working Paper/Pre-Print" OR "Literature Review" OR "Correction/Retraction" OR "Conference Paper" OR "Speech/Lecture" OR "Conference" OR "Statistics/Data Report" OR "Review" OR "Case Study" OR "Conference Proceeding" OR "Standard" OR "Article") AND la.exact("English" OR "Finnish") AND PEER(yes) AND ti(('Preterm born adults' OR preterm OR ELBW OR VPT OR extremely low birth weight OR SGA))<br>Source type<br>Books, Conference Papers & Proceedings, Dissertations & Theses, Encyclopedias & Reference Works, Evidence-Based Medical Resources, Scholarly Journals, Working Papers<br>Document type<br>Annual Report, Article, Book Chapter, Case Study, Conference, Conference Paper, Conference Proceeding, Correction/Retraction, Dissertation/Thesis, Government & Official Document, Letter To The Editor, Literature Review, Research Topic, Review, Speech/Lecture, Standard, Statistics/Data Report, Working Paper/Pre-Print<br>Language<br>English, Finnish

***An updated search was performed on 01-04-2021 to verify that no additional studies could be identified.

Supplemental Table S2. Reasons for exclusion from the systematic review.

Author	Title of Publication	Reason for Exclusion*
Aarnoudse-Moens et al. 2009	Meta-analysis of neurobehavioral outcomes in very preterm and/or very low birth weight children	Not assessed in adulthood
Aarnoudse-Moens et al. 2013	Executive Function and IQ Predict Mathematical and Attention Problems in Very Preterm Children	Not assessed in adulthood
Aasen et al. 2016	The relevance of the irrelevant: Attention and task-set adaptation in prematurely born adults	No ADHD symptom self-report, psychological interview or diagnosis
Adger-Antonikowski et al. 2009	A functionalist perspective of language ability and behavioral synchrony in the development of emotion regulation	Not assessed in adulthood
Anderson et al. 2003	ictorian Infant Collaborative Study G. Neurobehavioral outcomes of school-age children born extremely low birth weight or very preterm in the 1990s	Not assessed in adulthood
Anderson et al. 2011	Attention Problems in a Representative Sample of Extremely Preterm/Extremely Low Birth Weight Children	Not assessed in adulthood
Anderson et. Al 2003	Neurobehavioral Outcomes of school-age Children Born Extremelt Low Birthweight or very preterm in the 1990s	Not assessed in adulthood
Andreias et al. 2010	Neighborhood influences on academic achievement of extremely low birth weight children	Not assessed in adulthood
Asbury et al. 2006	Birthweight-discordance and difference in early parenting relate to monozygotic twin difference in behavior problems and academic achievement at age 7	Not assessed in adulthood
Astbury et al. 1985	Neonatal significance of behaviour in very low birthweight children	Not assessed in adulthood
Bachmann et al. 2021	Association of Preterm Birth with Prescription of Psychotropic Drugs in Adolescence and Young Adulthood	No ADHD symptom self-report, psychological interview or diagnosis—medication study
Beverly et al. 2008	Communication and academic challenges in early adolescence for children who have been adopted from the former Soviet Union	Not assessed in adulthood
Bhutta al.2002	Cognitive and behavioral outcomes of school-aged children who were born preterm: a meta-analysis.	Not assessed in adulthood
Bora et al. 2011	Emotional and behavioral adjustment of children born very preterm at earky school age	Not assessed in adulthood
Botellero et. Al 2016	Mental health and cerebellar volume during adolescence in very-low-birth-weight infants: a longitudinal study.	Mediator/Moderator/Mechanism study
Botting et al. 1997	Attention deficit hyperactivity disorders and other psychiatric outcomes in very low birthweight children at 12 years.	Not assessed in adulthood
Boulet et al. 2011	Birth weight and health and developmental outcomes in US children, 1997–2005	Not assessed in adulthood
Breslau et al. 1996	Psychiatric sequelae of low birth weight at 6 years of age.	Not assessed in adulthood
Breslau et al. 1996	Psychiatric sequele of low birth weight at 6 years of age.	Not assessed in adulthood
Brogan et al. 2014	Inattention in very preterm children: implications for screening and detection	Not assessed in adulthood
Brosschot et al. 1999	Processing bias in anxious subjects and repressors, measured by emotional Stroop interference and attentional allocation	No ADHD symptom self-report, psychological interview or diagnosis
Burnett et al. 2011	Prevalence of psychiatric diagnoses in preterm and full-term children, adolescents and young adults: a meta-analysis	Meta-analysis not specific to adulthood
Chu et al. 2012	The relationship between attention deficit hyperactivity disorder and premature infants in Taiwanese: a case control study	Not assessed in adulthood
Class et al. 2014	Fetal growth and psychiatric and socioeconomic problems: population-based sibling comparison	Population includes participants under 18

Conrad et al. 2010	Biological and environmental predictors of behavioral sequelae in children born preterm.	Not assessed in adulthood
Cooke et al. 1999	Cranial magnetic resonance imaging and school performance in very low birth weight infants in adolescence.	Not assessed in adulthood
Crea et al. 2014	Family environment and attention-deficit/hyperactivity disorder in adopted children: associations with family cohesion and adaptability	Not assessed in adulthood
Crump et al. 2010	Preterm birth and psychiatric medication prescription in young adulthood: A Swedish national cohort study	No ADHD symptom self-report, psychological interview or diagnosis—medication study
Dahl et al. 2006	Emotional, behavioral, social, and academic outcomes in adolescents born with very low birth weight.	Not assessed in adulthood
Dalziel et al. 2005	Psychological functioning and health-related quality of life in adulthood after preterm birth.	Includes participants born before 1970
Davis et al. 2007	Attention problems in very low birth weight preschoolers: are new screening measures needed for this special population	Not assessed in adulthood
de Kieviet et al. 2012	Attention problems of very preterm children compared with age-matched term controls at school-age	Not assessed in adulthood
de Zeeuw et al. 2012	Imaging gene and environmental effects on cerebellum in Attention-Deficit/Hyperactivity Disorder and typical development	Not assessed in adulthood
Delobel-Ayoub et al. 2006	Behavioral outcome at 3 years of age in very preterm infants : The EPIPAGE study	Not assessed in adulthood
D'Onofrio al.2013	Preterm Birth and Mortality and Morbidity A Population-Based Quasi-experimental Study	Population includes participants under 18
Duris et al. 2002	The incidence of attention deficit hyperactivity disorder among children born at low birth weight	Not assessed in adulthood
Elgen et al. 2002	Population based, controlled study of behavioural problems and psychiatric disorders in low birthweight children at 11 years of age.	Not assessed in adulthood
Elgen et al. 2012	Mental health at 5 years among children born extremely preterm: A national population-based study.	Not assessed in adulthood
Emond et al. 2006	Development and behaviour of low-birthweight term infants at 8 years in northeast Brazil: a longitudinal study	Not assessed in adulthood
Engeland et al. 2017	Preterm births and use of medication in early adulthood: a population-based registry study. Pharmacoepidemiology and Drug Safety	No ADHD symptom self-report, psychological interview or diagnosis—medication study
Eryigit-Madzwamuse	Attention problems in relation to gestational age at birth and smallness for gestational age	Not assessed in adulthood
Farooqi et al. 2007	Mental health and social competencies of 10- to12-year-old children born at 23 to 25 weeks of gestation inthe 1990s : a Swedish national prospective follow-up study.	Not assessed in adulthood
Farooqi et al. 2013	Behaviours related to executive functions and learning skills at 11 years of age after extremely preterm birth: A Swedish national prospective follow-up study.	Not assessed in adulthood
Fazio et al. 2008	Impact of pregnancy, delivery, and infancy complications on attention-deficit hyperactivity disorder	Not assessed in adulthood
Fernell et al. 2012	Preterm birth, ADHD and the ESSENCE in adult psychiatry	No ADHD symptom self-report, psychological interview or diagnosis
Fevang et al. 2016	Mental health in children born extremely preterm without severe neurodevelopmental difficulties	Not assessed in adulthood
Foulder-Hughes & Cooke 2003	Motor, cognitive, and behavioural disorders in children born very preterm.	Not assessed in adulthood
Franz et al. 2018	Attention-Deficit/Hyperactivity Disorder and Very Preterm/Very Low Birth Weight: A Meta-analysis	Meta-analysis not specific to adulthood
Frederick et al. 2012	Birth weight predicts scores on the ADHD Self-Report Scale and attitudes towards casual sex in college men: A short-term life history strategy?	Retrospective self-report of birthweight
Galera et al. 2011	Early risk factors for hyperactivity-impulsivity and inattention trajectories from age 17 months to 8 years	Not assessed in adulthood
Gardner et al. 2004	Behavioral and emotional adjustment of teenagers in mainstream school who were born before 29 weeks' gestation.	Not assessed in adulthood
Gatzke-Kopp et al. 2007	Direct and passive prenatal nicotine exposure and the development of externalizing psychopathology	Not assessed in adulthood

Giordano et al. 2014	PS-245 Is Early Detection Of Attention Problems In Preterm Children Possible? The Value Of Neuropsychological Assessment At Preschool Age	Not assessed in adulthood
Gray et al. 2004	Prevalence, stability, and predictors of clinically significant behavior problems in low birth weight children at 3, 5, and 8 years of age.	Not assessed in adulthood
Groen-Blokhuis et al. 2011	Evidence for a causal association of low birth weight and attention problems.	Not assessed in adulthood
Grunewaldt et al. 2014	Follow-up at age 10 years in ELBW children—functional outcome, brain morphology and results from motor assessments in infancy	Not assessed in adulthood
Hack et al. 2009	Behavioral outcomes of extremely low birth weight children at age 8 year	Not assessed in adulthood
Halmøy et al. 2012	Pre- and perinatal risk factors in adults with attention-deficit/hyperactivity disorder	No ADHD symptom self-report, psychological interview or diagnosis—medication study
Hanc et al. 2015	ADHD and overweight in boys: cross-sectional study with birth weight as a controlled factor	Not assessed in adulthood
Hanke et al. 2003	Preschool development of very low birth weight children born 1994–1995	Not assessed in adulthood
Hanke et al. 2003	Preschool development of very low birth weight children born 1994–1995	Not assessed in adulthood
Hatch et al. 2014	Associations between birth weight and attention−deficit/ hyperactivity disorder symptom severity: Indirect effects via primary neuropsychological functions	Not assessed in adulthood
Hatch et al. 2014	Associations between birth weight and attention−deficit/ hyperactivity disorder symptom severity: Indirect effects via primary neuropsychological functions	Not assessed in adulthood
Heinonen et al. 2011	Trajectories of growth and symptoms of attention-deficit/hyperactivity disorder in children: a longitudinal study	Not assessed in adulthood
Heinonen et al. 2018	Neurocognitive outcome in young adults born late-preterm	No ADHD symptom self-report, psychological interview or diagnosis
Hillie et a. 2008	Social lifestyle, risk-taking behavior and psychopathology in youngadults born very preterm or with a very low birthweight. The Dutch POPS study at 19 years of age.	No term or normal birth weight control group
Hillie et al. 2001	Behavioural problems in children who weigh 1000 g or less at birth in four countries.	Not assessed in adulthood
Hoff et. Al 2004	Behavioral and social development of children born extremely premature: 5-year follow-up	Not assessed in adulthood
Horwood et al. 1998	Cognitive, educational, and behavioural outcomes at 7 to 8 years in a national very low birthweight cohort	Not assessed in adulthood
Huang et al. 2012	Inattention and development of toddlers born in preterm and with low birth weight	Not assessed in adulthood
Hultman et al. 2007	Birth Weight and Attention-Deficit/Hyperactivity Symptoms in Childhood and Early Adolescence: A Prospective Swedish Twin Study	Not assessed in adulthood
Hutchinson et al. 2013	School-age outcomes of extremely preterm or extremely low birth weight children	Not assessed in adulthood
Indredavik et al. 2010	Perinatal Risk and Psychiatric Outcome in Adolescents Born Preterm With Very Low Birth Weight or Term Small for Gestational Age	Not assessed in adulthood
Indredavik et al. 2010	Perinatal Risk and Psychiatric Outcome in Adolescents Born Preterm With Very Low Birth Weight or Term Small for Gestational Age	duplicate
Jackson et al. 2015	Sibling differences in low birth weight, dopaminergic polymorphisms, and ADHD symptomatology: Evidence of GxE	Not assessed in adulthood
Jaekel et al. 2012	Poor attention rather than hyperactivity/impulsivity predicts academic achievement in very preterm and full-term adolescents	Not assessed in adulthood
James et al. 2018	Association of preterm birth with ADHD-like cognitive impairments and additional subtle impairments in attention and arousal malleability	No ADHD symptom self-report, psychological interview or diagnosis
Jaspers et al. 2013	Early childhood assessments of community pediatric professionals predict autism spectrum and attention deficit hyperactivity problems	Not assessed in adulthood
Johnson et al. 2010	Psychiatric Disorders in Extremely Preterm Children: Longitudinal Finding at Age 11 Years in the EPICure Study	Not assessed in adulthood
Johnson et. al 2016	Antecedents of Attention Deficit /Hyperacvtivity disorder symptoms in children born extremely preterm	Not assessed in adulthood

Johnson-Cramer et al. 1999	Assessment of school-aged children with comorbidity of attention deficit hyperactivity disorder and low birth weight classifications.	Not assessed in adulthood
Kadziela-Olech et al. 2005	The duration of breastfeeding and attention deficit hyperactivity disorder	Not assessed in adulthood
Kaufman al.1997	Schedule for Affective Disorders and Schizophrenia for School-Age Children-Present and Lifetime Version (K-SADS-PL): Initial Reliability and Validity Data	
Kelly et al. 2001	Birthweight and behavioural problems in children: a modifiable effect?	Not assessed in adulthood
Knopik et al. 2005	Contributions of parental alcoholism, prenatal substance exposure, and genetic transmission to child ADHD risk: A female twin study.	Not assessed in adulthood
Kreppner et al. 2001	Can inattention/overactivity be an institutional deprivation syndrome?	Not assessed in adulthood
Laerum al.2017	Psychiatric Disorders and General Functioning in Low Birth Weight Adults: A Longitudinal Study	
Lahat et al. 2014	ADHD among young adults born at extremely low birth weight: the role of fluid intelligence in childhood.	duplicate reporting of an already included study
Lahti et al. 2006	Small body size at birth and behavioural symptoms of ADHD in children aged five to six years.	Not assessed in adulthood
Langley et al. 2007	Effects of low birth weight, maternal smoking in pregnancy and social class on the phenotypic manifestation of Attention Deficit Hyperactivity Disorder and associated antisocial behaviour: Investigation in a clinical sample.	Not assessed in adulthood
Langley et al. 2010	Adolescent clinical outcomes for young people with attention-deficit hyperactivity disorder	Not assessed in adulthood
Leijon et al. 2016	Reading deficits in very low birthweight children are associated with vocabulary and attention issues at the age of seven.	Not assessed in adulthood
Levy-Schiff et al. 1994	Biological and environmental correlates of developmental outcome of prematurely born infants in early adolescence	Not assessed in adulthood
Linnet et al. 2006	Gestational age, birth weight, and the risk of hyperkinetic disorder	Not assessed in adulthood
Månsson et al. 2014	Behavioral outcomes at corrected age 2.5 years in children born extremely preterm.	Not assessed in adulthood
Mathewson et al. 2017	Mental Health of Extremely Low Birth Weight Survivors: A Systematic Review and Meta-Analysis	Meta-analysis not specific to adulthood
McCormick et al. 1990	Very low birth weight children: behavior problems and school difficulty in a national sample	Not assessed in adulthood
McGrath et al. 2005	Early Precursors Of Low Attention And Hyperactivity In A Preterm Sample At Age Four	Not assessed in adulthood
McNicholas et al. 2015	Mental health outcomes at age 11 of very low birth weight infants in Ireland	Not assessed in adulthood
McNicholas et al. 2015	Mental health outcomes at age 11 of very low birth weight infants in Ireland	Not assessed in adulthood
Melchior et al. 2015	Maternal tobacco smoking in pregnancy and children's socio-emotional development at age 5: The EDEN mother-child birth cohort study	Not assessed in adulthood
Mendez et al. 2011	Academic outcomes in Asian children aged 8-11 years with attention-deficit/hyperactivity disorder treated with atomoxetine hydrochloride	
Mick et al. 2002	Impact of low birth weight on attention-deficit hyperactivity disorder	Not assessed in adulthood
Minde et al. 1968	Studies on the hyperactive child.	Not assessed in adulthood
Mitchell et al. 1987	Clinical characteristics and serum essential fatty acid levels in hyperactive children	Not assessed in adulthood
Momany al.2018	A Meta-Analysis of the Association Between Birth Weight and Attention Deficit Hyperactivity Disorder	Meta-analysis not specific to adulthood
Momany et al. 2016	Sex moderates the impact of birth weight on childhood externalizing psychopathology	Not assessed in adulthood
Montes et al. 2009	Structural brain alterations in attention-deficit/hyperactivity disorder an update. First part	Mediator/Moderator/Mechanism study
Morales et al. 2013	Early precursors of low attention and hyperactivity in moderately and very preterm children at preschool age	Not assessed in adulthood
Murray et al. 2016	Are fetal growth impairment and preterm birth causally related to child attention problems and ADHD? Evidence from a comparison between high-income and middle-income cohorts	Not assessed in adulthood
Murray et. Al 2015	Sex differences in the association between foetal growth and child attention at age four: Specific vulnerability of girls	Not assessed in adulthood

Nadeau et al. 2001	Mediators of behavioral problems in 7-year-old children born after 24 to 28 weeks of gestation.	Not assessed in adulthood
Nadeau et al. 2003	Extremely Premature and Very Low Birthweight Infants: A Double Hazard Population?	Not assessed in adulthood
Nosarti et al. 2005	Hyperactivity in Adolescents Born Very Preterm Is Associated with Decreased Caudate Volume	Not assessed in adulthood
O'Callaghan et al. 1997	Biological predictors and co-morbidity of attention deficit and hyperactivity disorder in extremely low birthweight infants at school.	Not assessed in adulthood
Ocallaghan et al.	Biological and social predictors of Attention Deficit Disorder in extremely low birth weight children	Not assessed in adulthood
Offord et al. 1979	Delinquency and hyperactivity	Not assessed in adulthood
Orchinik et al. 2014	Symptoms of Attention Deficit Hyperactivity Disorder (ADHD) in Kindergarten as Predictors of Academic Progress in Extremely Low Birth Weight Children	Not assessed in adulthood
O'Shea et al. 2013	Extreme prematurity and attention deficit: epidemiology and prevention	In appropriate study design: review
Pal-de Bruin et al. 2015	Profiling the preterm or VLBW born adolescent; implications of the Dutch POPS cohort follow-up studies	Not assessed in adulthood
Parker et al. 2016	Prenatal smoking anbd childhood behavior problems: is the association mediated by birth weight?	Not assessed in adulthood
Perkinson-Gloor et al. 2015	The role of sleep and the hypothalamic-pituitary-adrenal axis for behavioral and emotional problems in very preterm children during middle childhood.	Not assessed in adulthood
Perricone et al. 2013	Neurodevelopmental outcomes of moderately preterm birth: precursors of attention deficit hyperactivity disorder at preschool age	
Pettersson et al. 2019	Association of Fetal Growth With General and Specific Mental Health Conditions	Population includes participants under 18
Pettersson et al. 2015	Birth weight as an independent predictor of ADHD symptoms: a within-twin pair analysis.	Not assessed in adulthood
Pettersson et al., 2015	Birth weight as an independent predictor of ADHD symptoms: a within-twin pair analysis.	duplicate
Poole et al. 2015	Childhood motor coordination and adult psychopathology in extremely low birth weight survivors	Not assessed in adulthood
Pyhälä et al. 2011	Neurocognitive abilities in young adults with very low birth weight	No ADHD symptom self-report, psychological interview or diagnosis
Razza et al. 2016	Links between motor control and classroom behaviors: Moderation by low birth weight	Not assessed in adulthood
Rice et al. 2010	The links between prenatal stress and offspring development and psychopathology: Disentangling environmental and inherited influences	Not assessed in adulthood
Rommel et al. 2019	Impairments in error processing and their association with ADHD symptoms in individuals born preterm	Mediator/Moderator/Mechanism study
Rommel et al. 2017	Altered EEG spectral power during rest and cognitive performance: a comparison of preterm-born adolescents to adolescents with ADHD	Not assessed in adulthood
Rommel et al. 2017	Association of Preterm Birth With Attention-Deficit/Hyperactivity Disorder-Like and Wider-Ranging Neurophysiological Impairments of Attention and Inhibition	No ADHD symptom self-report, psychological interview or diagnosis
Ross et al. 1992	Hand preference, prematurity and developmental outcome at school age	Not assessed in adulthood
Saigal et al. 2003	Psychopathology and social competencies of adolescents who were extremely low birth weight	Not assessed in adulthood
Samara et al. 2008	Pervasive behavior problems at 6 years of age in a total-population sample of children born at f25 weeks of gestation.	Not assessed in adulthood
Sammallahti et al. 2015	Infant Growth after Preterm Birth and Mental Health in Young Adulthood	No term or normal birth weight control group
Sasaluxnanon et al. 2005	Risk factor of birth weight below 2,500 grams and attention deficit hyperactivity disorder in Thai children	Not assessed in adulthood
Sato et al. 2004	Behavioral outcome including attention deficit hyperactivity disorder/hyperactivity disorder and minor neurological signs in perinatal high-risk newborns at 4–6 years of age with relation to risk factors	Not assessed in adulthood
Schmitz et al. 2006	Smoking during pregnancy and attention-deficit/hyperactivity disorder, predominantly inattentive type: a case-control study	Not assessed in adulthood
Schothorst et al. 2007	Psychiatric disorders and MND in non-handicapped preterm children	Not assessed in adulthood
Sciberras et al. 2011	Predictors of parent-reported attention-deficit/hyperactivity disorder in children aged 6–7 years: a national longitudinal study	Not assessed in adulthood

Scott et al. 2017	Neurocognitive Correlates of Attention-Deficit Hyperactivity Disorder Symptoms in Children Born at Extremely Low Gestational Age	Not assessed in adulthood
Scott et al. 2012	Behavior disorders in extremely preterm/extremley low birth weight children in kindergarten	Not assessed in adulthood
Scott et al. 2012	Behavior disorders in extremely preterm/extremley low birth weight children in kindergarten	duplicate
Sengupta et al. 2006	COMT Val108/158Met gene variant, birth weight, and conduct disorder in children with ADHD.	Not assessed in adulthood
Shariat et al. 2019	Association between small for gestational age and low birth weight with attention deficit and impaired executive functions in 3-6 years old children	Not assessed in adulthood
Shum et al. 2008	Attentional problems in children born very preterm or with extremely low birth weight at 7-9 years	Not assessed in adulthood
Simonds et al. 1980	Preterm birth, low birth weight, and hyperkinetic behavior in children.	Not assessed in adulthood
Smith et al. 2014	Angiogenic, neurotrophic, and inflammatory system SNPs moderate the association between birth weight and ADHD symptom severity	Not assessed in adulthood
St Sauver et al. 2004	Early life risk factors for attention-deficit/hyperactivity disorder: a population-based cohort study	Not assessed in adulthood
Stahlmann et al. 2009	Outcome of extremely premature infants at early school age: Health-related quality of life and neurosensory, cognitive, and behavioral outcomes in a population-based sample in northern Germany	Not assessed in adulthood
Sthernqvist & Svenningsen et al. 1999	Ten-year follow-up of children born before 29 gestational weeks: health, cognitive development, behaviour and school achievement.	Not assessed in adulthood
Sucksdorff et al. 2015	Preterm Birth and Poor Fetal Growth as Risk Factors of Attention-Deficit/Hyperactivity Disorder	Population includes participants under 18
Sullivan et al. 2012	17-year outcome of preterm infants with diverse neonatal morbidities: Part 1--Impact on physical, neurological, and psychological health status	Not assessed in adulthood
Sykes et al. 1997	Behavioural adjustment in school of very low birthweight children	Not assessed in adulthood
Szatmari et al. 1991	The Persistence of attention-Deficit Disorders among extremely low-birth-weight children (ELBW)	Not assessed in adulthood
Szatmari et al. 1990	Psychiatric disorders at five years among children with birthweights less than 1,000 g. A regional perspective.	Not assessed in adulthood
Szatmari et al. 1993	Psychopathology and adaptive functioning among extremely low birthweight children at eight years of age	Not assessed in adulthood
Taylor et al. 2019	Associations of attention deficit hyperactivity disorder (ADHD) at school entry with early academic progress in children born prematurely and full term controls	Not assessed in adulthood
Taylor et al. 1998	Attention deficits in children with 750 gm birth weight	Not assessed in adulthood
Taylor et al. 2000	Middle-school-age outcomes in children with very low birthweight	Not assessed in adulthood
Taylor et al. 2015	Persisting behavior problems in extremely low birth weight adolescents.	Not assessed in adulthood
Teplin et al. 1991	Neurodevelopmental, health, and growth status at age 6 years of children with birth weights less than 1001 grams.	Not assessed in adulthood
Torrioli et al. 2000	Perceptual-motor, visual and cognitive ability in very low birthweight preschool children without neonatal ultrasound abnormalities.	Not assessed in adulthood
Treyvaud et al. 2013	Psychiatric outcomes at age seven for very preterm children: rates and predictors	Not assessed in adulthood
Vaag et al. 2019	Psychoeducational groups versus waitlist in treatment of attention-deficit hyperactivity/impulsivity disorder (ADHD) in adults: a protocol for a pilot randomized waitlist-controlled multicenter trial.	Not assessed in adulthood
Van den Bergh et al. 2004	High Antenatal Maternal Anxiety Is Related to ADHD Symptoms, Externalizing Problems, and Anxiety in 8- and 9-Year-Olds	Not assessed in adulthood
Van Hus et al. 2014	Motor impairment in very preterm-born children: links with other developmental deficits at 5 years of age	Not assessed in adulthood
van Mil et al. 2015	Low and high birth weight and the risk of child attention problems	Not assessed in adulthood
Wagner et al. 2009	The limited effects of obstetrical and neonatal complications on conduct and attention-deficit hyperactivity disorder symptoms in middle childhood	Not assessed in adulthood
Waldie et al. 2017	Dopamine transporter (DAT1/SLC6A3) polymorphism and the association between being born small for gestational age and symptoms of ADHD	Not assessed in adulthood

Walshe et al. 2008	Psychiatric disorder in young adults born very preterm: role of family history.	No ADHD symptom self-report, psychological interview or diagnosis
Wiles et al. 2006	Fetal growth and childhood behavioral problems: results from the ALSPAC cohort	Not assessed in adulthood
Williams et al. 2010	hort and long-term effects of birth weight and neonatal medical complications on<br>children’s emotional and behavioral outcomes	Not assessed in adulthood
Wilson-Ching et al. 2013	Attention Difficulties in a Contemporary Geographic Cohort of Adolescents Born Extremely Preterm/Extremely Low Birth Weight	Population includes participants under 18
Wolke et al .2019	The Life Course Consequences of Very Preterm Birth	In appropriate study design: review
Woodward et al. 2017	Preschool self-regulation predicts later mental health and educational achievement in very preterm and typically developing children.	Not assessed in adulthood
Woodward et al. 2017	Preschool self-regulation predicts later mental health and educational achievement in very preterm and typically developing children.	Not assessed in adulthood
Yang et al.2015	Psychiatric Diagnoses, Emotional-Behavioral Symptoms and Functional Outcomes in Adolescents Born Preterm With Very Low Birth Weights	Not assessed in adulthood
Yang et al. 2015	Psychiatric diagnoses, emotional-behavioral symptoms and functional outcomes in adolescents born preterm with very low birth weights	Not assessed in adulthood
Young al.2019	Adult Mental Health Outcomes of Preterm Survivors Experiencing Suicidal Ideation in Adolescence	Not assessed in adulthood

**Studies may have more than one reason for exclusion, however, only one reason is indicated here*

Supplemental Table S3. Attention deficit / hyperactivity (ADHD) disorders symptoms scales and raw ADHD symptoms scores in preterm and term-born controls by cohort studies included in the Individual Participant Data meta-analysis.

Scale	Cohort	Questionnaire	Preterm		Term Controls		P value
			M	*SD*	*M*	*SD*	
ADHD Total Score	*AYLS*[1]	ASEBA ASR	4.70	4.18	4.93	4.30	.517
		ASRS	21.85	10.62	22.55	10.40	.434
	BLS[2]	ASEBA-YASR	1.69	1.75	1.71	1.49	.854
		ARS DSM III/IV	1.73	2.37	1.89	2.20	.457
	EPICure[3]	ASEBA ASR	6.35	5.30	4.25	4.05	.013
		ARS DSM III/IV	3.29	3.66	2.29	2.94	.089
	ESTER[4]	ASEBA ASR	4.80	3.85	4.43	3.32	.183
		ASRS	21.80	8.33	21.83	7.79	.964
	HeSVA[5]	ASEBA ASR	5.16	4.53	5.61	4.24	.466
		ASRS	21.90	9.75	23.17	9.07	.336
	NTNU LBW Life[6]	ASEBA ASR	5.58	4.00	3.35	3.89	.001
		ASRS	21.45	10.62	18.59	8.74	.081
	NZVLBW[7]	ARS DSM III/IV	1.60	2.23	1.19	1.69	.162
	VICS[8]	CADS	12.74	10.09	14.85	10.26	.193
Hyperactivity/Impulsivity Score	*AYLS*	ASEBA ASR	1.90	2.01	2.12	2.23	.237
		ASRS	9.81	5.56	10.35	5.75	.264
	BLS	ASEBA-YASR	.80	.232	.94	.94	.135
		ARS DSM III/IV	1.09	1.50	1.38	1.56	.035
	EPICure	ASEBA ASR	2.84	2.70	2.29	2.35	.216
		ARS DSM III/IV	1.68	1.93	1.59	1.65	.758
	ESTER	ASEBA ASR	2.03	1.97	1.90	1.84	.372
		ASRS	9.86	4.71	10.02	4.51	.659
	HeSVA	ASEBA ASR	2.27	2.41	2.51	2.12	.459
		ASRS	9.77	5.41	10.88	4.96	.125
	NTNU LBW Life	ASEBA ASR	3.17	2.47	1.91	2.26	.002
		ASRS	9.69	5.11	8.52	4.44	.180
	NZVLBW	ARS DSM III/IV	.90	1.32	.78	1.07	.517
	VICS	CADS	5.98	5.44	6.46	5.42	.576
Inattention Score	*AYLS*	ASEBA ASR	2.81	2.67	2.80	2.65	.948
		ASRS	12.16	5.76	12.19	5.69	.763
	BLS	ASEBA-YASR	.88	1.19	.78	.90	.285
		ARS DSM III/IV	.65	1.28	.51	1.07	.221
	EPICure	ASEBA ASR	3.52	3.09	1.96	2.07	.001
		ARS DSM III/IV	1.59	2.12	.71	1.60	.009
	ESTER	ASEBA ASR	2.77	2.44	2.53	2.05	.171
		ASRS	11.95	5.19	11.80	4.37	.695
	HeSVA	ASEBA ASR	2.89	2.73	3.10	2.73	.579
		ASRS	12.14	5.93	12.29	5.10	.844
	NTNU LBW Life	ASEBA ASR	2.42	2.37	1.44	2.07	.010
		ASRS	11.75	6.20	10.06	5.05	.075
	NZVLBW	ARS DSM III/IV	.70	1.21	.41	.90	.061
	VICS	CADS	6.77	5.68	8.38	5.84	.076
Attention Score	*AYLS*	ASEBA ASR	6.59	4.90	6.89	4.88	.471
	BLS	ASEBA YASR	2.78	2.53	2.47	2.02	.144
	EPICure	ASEBA ASR	7.59	5.82	4.45	4.17	.001
	ESTER	ASEBA ASR	6.74	4.56	6.27	3.65	.145
	HeSVA	ASEBA ASR	7.53	5.09	7.97	4.88	.529
	NTNU LBW Life	ASEBA ASR	7.03	4.77	4.62	4.37	.002
	NZ VLBW	NA	-		-		-
	VICS	NA	-		-		-

**Not Applicable as the scale available does not have a comparable attention problems measure*

[1]Arvo Ylppö Longitudinal Study (Uusimaa, Finland, born 1985)

[2]Bavarian Longitudinal Study, also known as BEST (Bayerische Entwicklungsstudie) (Germany, born 1985)

[3]EPICure Cohort (UK & The Republic of Ireland, born 1995)

[4][Preterm Birth and Early Life Programming of Adult Health and Disease] Ennenaikainen syntymä, raskaus ja lapsen terveys aikuisiässä, Northern Finland, born 1985-1989)

[5]Helsinki Study of Very Low Birth Weight Adults (Helsinki, Finland, born 1978-1985)

[6]Norges Teknisk-Naturvitenskapelige Universitet [Norwegian University of Science and Technology] Low Birth Weight Life (Trondheim, Norway, born 1986-1988)

[7]New Zealand Very Low Birth Weight 1986 Cohort (New Zealand, born 1986)

[8]Victorian Infant Collaborative Study (Victoria, Australia, born 1991-1992)

Supplemental Table S4. Newcastle-Ottawa-Scale quality of evidence assessment criteria.	
**Quality assessment criteria**	**Acceptable Criteria (★)**
**Selection**	★★★★
Representativeness of the exposed cohort?	Representative of adults preterm born (<37 gestational weeks) or very low birth weight(<1500 g).
Selection of the non-exposed cohort?	Drawn from same community as exposed cohort.
Ascertainment of exposure	Gestational age and/or birthweight from medical records or birth register. Not parent or self-reported.
Demonstration that outcome of interest was not present at start of study	Longitudinal follow-up study starting from birth. Outcome could not have occurred before birth.
**Comparability**	★★
Comparability of cohorts on the basis of the design or analysis	Study controls for familial confounding i.e. sibling analysis, parental ADHD, etc…
Study controls or otherwise takes into account for at least 3 additional risk factors?	Study controls for (or otherwise takes statistically into account) sex, age, family Socioeconomic Status, parental education, maternal age at birth.
**Outcome**	★★★
Assessment of outcome	Diagnostic Interview or medical record diagnosis of ADHD.
Was follow-up long enough for outcomes to occur	All individuals studied followed to adulthood---at least 18 years of age.
Adequacy of follow up of cohorts	Complete follow-up, or subjects lost to follow-up unlikely to introduce bias (>50% follow up).
**Overall quality score (maximum=9)**	

Supplemental Table S5. Number of participants according to the degree of preterm birth in cohort studies included in the Individual-Participant-Data meta-analysis in the population-based register-linkage study.

**Cohort**	**Extremely Preterm** <28+0 gestational weeks + days			**Very Preterm** 28+0 to 31+6 gestational weeks + days			**Moderate to Late Preterm** 32+0 to 36+6 gestational weeks + days			**Term Controls** 37+0 to 41+6 gestational weeks + days		
	**N**	**% within cohort**	**% contributed by cohort to the IPD meta-analysis**	**N**	**% within cohort**	**% contributed by cohort to the IPD meta-analysis**	**N**	**% within cohort**	**% contributed by cohort to the IPD meta-analysis**	**N**	**% within cohort**	**% contributed by cohort to the IPD meta-analysis**
All Clinical Cohorts	319	10.6	NA	489	16.2	NA	577	19.1	NA	1513	52.5	NA
AYLS[1]	6	.6	1.9	20	2.2	4.1	145	16.1	25.1	727	81.0	48.1
BLS[2]	16	3.5	5.0	170	37.4	34.8	48	10.6	8.3	220	48.5	14.5
EPICure[3]	117	69.6	36.7	0	0	0	0	0	0	51	30.6	3.4
ESTER[4]	8	1.2	2.5	51	7.4	10.4	313	45.6	54.2	315	45.9	20.8
HeSVA[5]	31	14.9	9.7	63	30.3	12.9	17	8.2	2.9	97	4.6	6.4
NTNU LBW Life[6]	18	12.7	5.6	31	21.8	6.4	11	8.2	1.9	82	57.8	5.4
NZ VLBW[7]	57	24.8	17.9	134	58.3	27.4	39	17.0	6.8	69	23.1	4.6
VICS[8]	66	40.7	20.7	20	12.3	4.1	4	2.5	0.7	72	44.4	4.8
Population-based register-linkage Study[9]	973	0.13	NA	3233	0.45	NA	32756	4.54	NA	684730	94.8	NA

[1]Arvo Ylppö Longitudinal Study (Uusimaa, Finland, born 1985)
[2]Bavarian Longitudinal Study (Bayerische Entwicklungsstudie) (Germany, born 1985)
[3]EPICure 1995 cohort(UK & The Republic of Ireland, born 1995)
[4]Ennenaikainen syntymä, raskaus ja lapsen terveys aikuisiässä [Preterm Birth and Early Life Programming of Adult Health and Disease](Northern Finland, born 1985-1989)
[5]Helsinki Study of Very Low Birth Weight Adults (Helsinki, Finland, born 1978-1985)
[6]Norges Teknisk-Naturvitenskapelige Universitet [Norwegian University of Science and Technology] Low Birth Weight Life (Trondheim, Norway, born 1986-1988)
[7]New Zealand Very Low Birth Weight 1986 Cohort(New Zealand, born 1986)
[8]Victorian Infant Collaborative Study (Victoria, Australia, born 1991-1992)
[9]All live births in Finland between 1/1/21987-31/12/1998
*NA (not applicable)

Supplemental Table 6. Associations between covariates and attention deficit / hyperactivity disorder (ADHD) symptoms in the Individual Participant Data meta-analysis of cohorts and ADHD diagnosis in the population-based register-linkage study in adulthood.

	Individual Participant Data meta-analysis								Population-based register-linkage study			
	ADHD symptoms Z score				ADHD above vs below clinical cutoff				ADHD diagnosis yes versus no			
Covariates	Estimate	95% CI		p	OR	95% CI		p	RR	95% CI		p
Parental education												
High (ISCED 6-8)	Ref				Ref				Ref			
Medium (ISCED 3-5)	0.03	-0.04	0.10	0.40	1.24	0.96	1.59	0.10	1.06	0.99	1.14	0.14
Low (ISCED 0-2)	0.20	0.09	0.31	0.0004	1.82	1.27	2.58	0.001	1.64	1.49	1.81	<0.001
Male versus. female	-0.14	-0.21	-0.08	<0.001	0.78	0.62	0.97	0.03	1.71	1.61	1.82	<0.001
Age in adulthood (years)	0.00	-0.01	0.01	0.96	0.94	0.87	1.01	0.11	1.01	1.00	1.02	0.02

Estimate refers to mean difference in ADHD symptoms between categorical covariates and standard deviation unit change per each unit increase in the covariate. OR refers to odds ratio, RR to relative risk, 95% CI to 95% Confidence Interval and ISCED to the International Standard Classification of Education.

Supplemental Table S7. Associations between gestational age and birth weight categories with attention deficit / hyperactivity disorder (ADHD) symptoms in the Individual Participant Data meta-analysis of cohorts and ADHD diagnosis in the population-based register-linkage study in adulthood.

	Individual Participant Data meta-analysis								Population-based register-linkage study ADHD diagnosis yes versus no			
	ADHD symptoms Z score				ADHD above versus below clinical cutoff							
**Predictors**	**Estimate**	**95% CI**		**p**	**OR**	**95% CI**		**p**	**RR**	**95% CI**		**p**
**Term versus preterm categories**												
Term (37+0 – 41+6 wks+days)	Ref				Ref				Ref			
Moderate to late preterm (32+0 – 36+6 wks+days)	0.00	-0.09	0.09	0.99	1.07	0.78	1.46	0.68	1.13	1.00	1.29	0.06
Very preterm (28+0 – 31+6 wks+days)	0.00	-0.10	0.09	0.95	0.90	0.63	1.28	0.56	2.37	1.80	3.12	<0.001
Extremely preterm (<28+0 wks + days)	0.01	-0.12	0.14	0.88	1.53	1.00	2.35	0.05	1.70	0.94	3.06	0.08
**Term versus preterm not small-for-gestational age and small-for-gestational-age**												
Term (37+0-41+6 wks+days)[1]	Ref				Ref				Ref			
Preterm (<37 wks+days) and not small-for-gestational-age (≥-2SD)	0.00	-0.07	0.08	0.93	1.06	0.83	1.36	0.64	1.20	1.06	1.35	0.002
Preterm (<37 wks+days) and small-for-gestational-age (<-2SD)	-0.01	-0.20	0.17	0.92	1.10	0.58	2.07	0.78	2.81	1.85	4.25	<0.001

[1]Term category includes both small- and appropriate for gestational age birth weight. Estimate refers to mean difference in ADHD symptoms, OR to odds ratio, RR to relative risk, and 95% CI to 95% Confidence Interval. All associations are adjusted for participant's sex, age (in adulthood follow-up in the meta-analysis of cohorts and age at death, emigration or end of follow-up [31/12/2016] in the register-linkage study) and parental education (of either parent in the meta-analysis of cohorts and of maternal in the register-linkage study).
